# Resonant Eddy Current Sensor Design for Corrosion Detection of Reinforcing Steel

**DOI:** 10.3390/s24134211

**Published:** 2024-06-28

**Authors:** Upeksha Chathurani Thibbotuwa, Ainhoa Cortés, Aurora María Casado, Andoni Irizar

**Affiliations:** 1 CEIT-Basque Research and Technology Alliance (BRTA), Manuel Lardizabal 15, 20018 Donostia-San Sebastián, Spain; airizar@ceit.es; 2 Department of Electronics & Communications, Tecnun, Universidad de Navarra, Manuel Lardizabal 13, 20018 Donostia-San Sebastián, Spain; 3 ACCIONA Construction Innovation Technology Division, C/Valportillo II n◦ 8, 28108 Alcobendas, Spain; auroramaria.casado.barrasa@acciona.com

**Keywords:** corrosion detection, eddy current, LC resonator, reinforcing bar, single frequency

## Abstract

This paper introduces an LC resonator-based single-frequency eddy current (EC) sensor designed for corrosion detection in reinforcing bars (rebars) embedded within concrete structures. The work addresses the challenges of the limited detection ranges and reduced sensitivity over longer distances, prevalent in current EC sensor applications. The sensor development process involved a systematic experimental approach to carefully selecting each parameter in the LC resonator. The sensor design aimed to assess the condition of the rebar from a distance of up to 5–6 cm outside the concrete and provide insights into different corrosion levels. By examining the characteristics of the inductors, the parallel resistance $R_{p}$ of the eddy current coil was identified as a key parameter reflecting the corrosion conditions in the rebar. The relationship between the $R_{p}$ fluctuations and temperature variations was investigated, with the data indicating that an approximately 155 Ω variation can be expected per 1 °C change within the temperature range of 20–25 °C, allowing for temperature compensation if necessary. Subsequently, the sensor’s performance was evaluated by placing a rebar within a concrete block, where controlled mechanical degradation cycles were applied to simulate uniform corrosion in the rebar. The experimental results show that our EC sensor can detect material loss around the rebar with accuracy of approximately 0.17 mm.

## 1. Introduction

Reinforced concrete structures are susceptible to corrosion, a natural process that initiates when the steel rebar within the concrete begins to corrode. Corrosion in reinforced concrete occurs when the protective layer around the steel reinforcement is compromised, making the steel vulnerable to deterioration. The consequences of corrosion can be severe, leading to a reduced load-bearing capacity, structural instability, and potentially catastrophic failures if left untreated. Given the hidden nature of corrosion within concrete structures and the potential for rapid deterioration once visible, early detection is crucial for timely maintenance and in preventing costly damage. However, corrosion detection in reinforced concrete structures at an early stage presents challenges due to the concealed nature of the reinforcement bars and the complex interaction between the corrosion process and the concrete matrix. These challenges become more pronounced in offshore environments due to the combined environmental effects of moisture, saltwater, and oxygen, which accelerate the corrosion process. Moreover, offshore settings introduce further complications in inspection and maintenance activities due to restricted accessibility, adverse operational conditions, technological constraints, and costs.

Focusing on the reinforcing bars (rebars) within concrete elements, corrosion in these rebars is essentially an electrochemical process that occurs through a series of chemical reactions involving the steel (i.e., reinforcing steel) and the moisture and oxygen present in the concrete environment (i.e., concrete pore solution). Once the corrosion of the reinforcement steel in a reinforced concrete structure begins, it tends to advance at a relatively constant rate [1], and, with time, the corrosion products (iron oxides and hydroxides) are deposited in the limited space of the concrete surrounding the steel. This will cause expansive stresses, leading to cracks and spalling the concrete cover.

Reinforced concrete (RC) structures can experience both uniform and non-uniform corrosion, depending on various factors and the specific conditions to which they are exposed. Uniform corrosion typically occurs under relatively favorable environmental conditions, where the concrete structure is subjected to a consistent and mildly corrosive environment [2]. Uniform corrosion in atmospherically exposed RC structures is influenced primarily by the availability of water and the pore structure at the steel–concrete interface. On the other hand, non-uniform corrosion is characterized by the uneven or localized corrosion (such as pitting) of the steel reinforcement. This type of corrosion is often more concerning and damaging than uniform corrosion. Non-uniform corrosion can result from various factors, including localized exposure, the presence of chlorides, the quality of the concrete mix (concrete properties), dissimilar metals, and local environmental factors such as the micro-climatic conditions—areas with standing water or exposure to pollutants can contribute to localized corrosion [2,3]. Additionally, the temperature and humidity can cause both uniform and non-uniform corrosion in reinforced concrete (RC) structures, primarily through their influence on the moisture content within the concrete and the mean temperature of the concrete structure [4].

Non-destructive testing (NDT) techniques are widely employed to investigate the mechanisms and kinetics of corrosion in steel embedded within concrete structures. These NDT methods are invaluable in evaluating the condition of reinforcing steel bars (rebars) without exerting any destructive impact on the test object. For long-term corrosion monitoring systems, it is recommended to employ technological approaches that are both NDT and non-invasive, where the non-invasiveness means that the testing method does not alter the material condition due to the technical approach that we use. Some of these techniques focus on determining the concrete properties, while others focus on the rebar properties for corrosion detection.

- Concrete Property Evaluation: This primarily focuses on assessing the properties of the concrete itself and helps to determine how susceptible the concrete is to corrosion. It may include measures of the concrete’s porosity, moisture content, and permeability and the presence of cracks or defects. Understanding these concrete properties is crucial because they influence the ingress of aggressive substances, such as chloride ions or carbon dioxide, which can trigger steel corrosion within the structure. Some examples are surface potential (SP) measurements, concrete resistivity measurement, infrared thermography, etc.
- Rebar Property Analysis: On the other hand, some techniques are more focused on evaluating the properties of the reinforcing steel bars (rebars) within the concrete. These methods aim to assess the condition of the rebar, such as its corrosion rate, corrosion products, and thickness loss and the degree of steel passivation. Understanding the rebar properties is essential because it allows us to directly assess the corrosion condition of the rebars. Some examples are half-cell potential (HCP) measurement, open circuit potential (OCP) measurement, linear polarization resistance (LPR) measurement, ultrasonic testing (UT), electromagnetic testing, etc.

In this context, the corrosion detection sensors employed in NDT techniques play a key role in the timely assessment of corrosion within reinforced concrete by providing the early detection of damage [5]. As these sensors measure parameters indicative of corrosion, the resolution and sensitivity that they offer are important in accurately reflecting the current state of corrosion. However, these sensors have inherent limitations based on their sensing principles, which may become particularly challenging based on the application requirements. For example, when rebars embedded in concrete are inspected from the outer surface, the use of ultrasound sensors presents more challenges compared to that of electromagnetic sensors. This is mainly because ultrasound sensors require extra preparation to establish good-quality contact with the testing concrete surface; furthermore, high-frequency ultrasound waves are significantly attenuated in thicker concrete structures, leading to the potential loss of critical information. Additionally, the internal structure of concrete, with its varying density and the presence of aggregates, can also cause the scattering and reflection of the ultrasound waves, further complicating data interpretation. In contrast, electromagnetic sensors are primarily responsive to conductive materials. Provided that the magnetic field that they generate is strong enough, they can be more effective in assessing only the rebar within the concrete, as they are less influenced by the surrounding non-conductive matrix.

The assessment of corrosion in reinforced concrete structures has been the subject of extensive research, leading to the development of various sensor-based techniques. Numerous reviews in this field offer insights into the different assessment methods [3,6,7]. Since the corrosion of reinforcement steel in concrete is an electrochemical process, electrochemical methods have traditionally been the most prevalent techniques used to assess and determine the corrosion rate in concrete structures.

However, there is a growing trend towards evaluating corrosion in reinforced concrete by examining its physical properties. These physical property assessment techniques offer several advantages, which include the provision of immediate results during testing (measurement speed), allowing for real-time assessments, offering a direct and multi-parameter approach to detect various corrosion-related issues, and including the thickness loss, reduced adhesion at the steel/concrete boundary, and cracks due to corrosion product accumulation. While electrochemical methods remain important, the industry increasingly recognizes the benefits of physical property assessment techniques for more effective and timely corrosion monitoring practices.

The approach proposed in this paper focuses on assessing the physical parameters of rebars for corrosion monitoring. Specifically, this solution aims to monitor concrete offshore structures (e.g., breakwater) to protect photovoltaic (PV) offshore platforms. Within this framework, our efforts are dedicated to developing a robust and cost-effective corrosion monitoring sensor node to be deployed on offshore breakwater.

## 2. Sensor Development: Corrosion Detection in Rebars by Physical Property Assessment

Critical factors for the selection of an appropriate sensing principle include ensuring the sensitivity to detect developing corrosion at earlier stages and the selectivity to distinguish corrosion from other interfering signals, such as environmental noise, physical disturbances, temperature variations, and humidity changes. Moreover, given the objectives of the application, it is important to consider the required measurement depth and ensure compatibility with the intended sensor integration and data collection infrastructure. Moreover, in offshore environments, the sensor must be durable, withstanding harsh marine environmental conditions, and provide reliable data. The practical usability of such sensors is underpinned by straightforward calibration procedures, user-friendly interfaces, and sophisticated algorithms for accurate data analysis. Lastly, regulatory compliance, cost-effectiveness, and sustainability considerations are also important in successfully implementing these sensors in the construction and inspection industry.

In the context of evaluating steel bars within reinforced concrete structures, a non-destructive technique such as eddy current testing (ECT), which is based on electromagnetic induction, enables the assessment of the reinforcement’s condition without requiring invasive procedures. This method involves generating localized circular currents, known as eddy currents, within the steel bars through the application of electromagnetic fields. The subsequent analysis of variations in these currents helps in detecting discontinuities or changes in the material properties of the steel bars, such as potential corrosion or cracks. When applied to offshore concrete structures, electromagnetic NDT methods offer several advantages. These include the elimination of direct physical contact with the rebars or concrete structure, which is particularly beneficial in environments where access may be constrained. Additionally, these methods generally require minimal surface preparation, which can greatly reduce the inspection preparation times by eliminating the need for coating removal or extensive cleaning beforehand. This facilitates a rapid inspection process, enabling quick measurements and the potential for the real-time monitoring of structural health. Furthermore, electromagnetic NDT methods can provide quantitative data on the condition of the rebars, such as their level of corrosion or the loss of the cross-sectional area, which is important for the effective maintenance and extension of the service life of offshore structures.

In our application, we anticipate the development of a compact, low-power, and cost-effective sensor node that can be used to assess the condition of rebar corrosion from the exterior of a concrete structure. Given these specifications, eddy current NDT emerges as a particularly fitting technique for the detection of corrosion in reinforcements. Its non-destructive nature and its ability to detect surface and sub-surface flaws in conductive materials embedded in concrete, without requiring direct contact with the surface of the concrete, is very suitable for the target application. Moreover, it enables the low-cost sensing of conductive targets in the presence of dust and moisture, making this technology reliable in harsh environments.

This area of study aims to explore the various aspects of the eddy current technique concerning corrosion-induced degradation in concrete-based infrastructure, including the following.

- Eddy current principles: studies have explored the fundamental principles of eddy current testing, which involves inducing electromagnetic currents in the steel bars and analyzing the changes in the eddy current properties caused by corrosion.
- Sensor development: studies have focused on developing specialized sensors and probes capable of generating and measuring the eddy currents in steel bars.
- Corrosion detection: research has aimed at refining the sensitivity of eddy current techniques to detect and quantify corrosion in steel reinforcements.
- Data analysis: studies have developed algorithms and data analysis methods to interpret the signals generated by eddy current testing.
- Field applications: field trials and case studies have been used to evaluate the effectiveness of eddy current-based corrosion monitoring in real-world scenarios.
- Long-term corrosion monitoring: given the importance of long-term corrosion monitoring in infrastructure maintenance, studies have explored strategies for the continuous and remote monitoring of reinforced steel bars using eddy current technology.

### State of Art: Eddy-Current-Based Corrosion Detection

Eddy current (EC) probe design is essential in obtaining good results in eddy current testing. There are different types of eddy current probes available, such as single coil probes, eddy current array probes, rotating probes, bobbin probes, flexible probes, etc. A review of the eddy current testing probes for carbon steel pipeline assessment inspection is presented in [8]. Depending on the probe design, eddy current testing can be performed using either a single frequency or multiple frequencies. Single-frequency eddy current probes are designed to operate at a fixed, predetermined frequency during the inspection process. These probes are particularly quick to identify the presence or absence of defects or for thickness estimation [9]. Multi-frequency eddy current sensors use several frequencies to assess the test object. A multi-frequency approach in the estimation of the thickness for a given material is discussed in [10]. This work analyzes the change in the peak frequency of the imaginary part of the inductance using a non-magnetic metallic plate.

The effectiveness of the eddy current sensor is closely related to the precise design of the inductor coil, as it plays a crucial role in determining their performance and functionality. The coil’s geometry, encompassing factors like its shape and size, directly impacts the magnetic field and its interaction with the target material [11,12].

An LC tank-based eddy current corrosion detection system is a cutting-edge technology that employs an LC (inductance–capacitance) resonator as its measurement probe. In eddy current NDT-based applications, an LC resonator has been adapted to serve as a single-frequency probe in eddy current testing. In general, an LC resonator is designed to resonate at a pre-defined frequency, and this fixed resonance frequency is utilized as the probing frequency during the inspection.

In the literature, there are several research works found based on ECT with LC resonators, considering different scopes. The authors of [13] propose a method to characterize the conductivity measurement of non-ferromagnetic metallic materials using an LC probe. The measurement parameters—the resonant frequency and series resonant resistance of the coil—are modeled as a function of the conductivity and the lift-off effect, where an experiment is carried out to test samples with different electrical conductivity values. In certain configurations, eddy current sensors are designed with separate transmitter and receiver coils, as discussed in the work by Andringa (2005) [14]. This work presents a prototype of a sensor with an LC resonator developed for reinforced concrete that consists of a reader and LC resonator circuit, which are magnetically coupled. A bare steel wire is connected to the LC resonator circuit to act as a transducer by exposing it to the same concrete environment as the reinforcing steel. The phase response of the capacitor of the resonator circuit is measured to read the corrosion activity of this bare steel wire. In this study, instead of directly measuring the corrosion condition of the rebars, they assess it indirectly by subjecting a bare steel wire to the concrete environment. A self-frequency conversion measurement system is presented in [15] based on an LC resonator for fiber-reinforced plastics in its conventional frequency range of operation of 1 kHz to 1 MHz. The measurement system is designed so that the resonant frequency is not fixed, allowing it to change yet keeping it always at a resonant level. A controlled oscillator with a feedback loop is used to detect the change in the resonant state. In [16], a three-point capacitive resonance circuit is presented for defect inspection in carbon-fiber-reinforced polymer testing based on the change in the resonance frequency of the output signal.

Existing EC sensors often encounter challenges related to their restricted detection ranges and low sensitivity, particularly when operating over longer distances. To our knowledge, achieving reliable detection at distances around 5 cm is uncommon in current applications, as eddy currents are typically utilized for shorter-distance inspections. Addressing this challenge, our research focuses on developing an EC sensor designed to detect different corrosion levels in reinforcing bars embedded in concrete and operates effectively over longer distances. For this task, we use an experimental methodology focused on sensitivity and robustness, quantified corrosion measures, specifically target applications where precise monitoring at greater distances is essential. Our research represents a significant step forward in EC sensor technology, offering a specialized solution for reinforced concrete structures that meets the specific demands of corrosion detection from comparatively greater distances.

## 3. Eddy Current Testing Principle

Eddy current testing (ECT) for corrosion detection in rebars operates on the principle of electromagnetic induction. When an alternating current is passed through a coil or probe, it generates an alternating magnetic field around it. If an electrically conductive material is in the vicinity of this electromagnetic field, surface currents known as eddy currents are induced within the material, according to Faraday’s law of electromagnetic induction. The magnitude and distribution of the eddy currents are influenced by the distance, size, and composition of the conductor. These induced eddy currents, in turn, create an electromagnetic field opposing the primary magnetic field produced by the coil. The interaction between these fields induces a back electromotive force (EMF) in the coil, causing a measurable change in the coil’s impedance.

When corrosion or defects are present within the rebar, they lead to variations in its material properties, particularly its magnetic permeability and electrical conductivity. These variations lead to inconsistencies in the eddy current patterns during the ECT process. Corrosion typically results in lower electrical conductivity because it changes the metal into non-conductive corrosion products. This change is reflected as an alteration in the impedance of the eddy currents. In addition, the magnetic permeability of the rebar is influenced by the presence of corrosion. The development of corrosion products or the introduction of cracks can disrupt the uniform magnetic properties of the steel. As a result, the interaction of the probe’s magnetic field with the rebar is changed, which in turn affects the secondary magnetic field produced by the eddy currents. By measuring and analyzing these disturbances, ECT can assess the condition of the rebar, detecting variations such as corrosion-induced pitting or thinning.

One major advantage of this technique is its ability to function without requiring direct contact with the test object, making it appropriate for online and in-service applications. However, for a long-term, reliable EC sensor, further studies are necessary to address challenges such as calibration complexities, the accurate quantification of corrosion, robust measurements, and the mitigation of environmental factors that affect the measurement accuracy.

### Single-Frequency Eddy Current Sensor with an LC Resonator

LC resonators can function as eddy current probes operating at a single frequency by configuring them within a resonant LC circuit arrangement. This arrangement consists of a coil (inductor, *L*) and a capacitor (C) connected in parallel, forming a resonant LC circuit.

**Figure 1:**
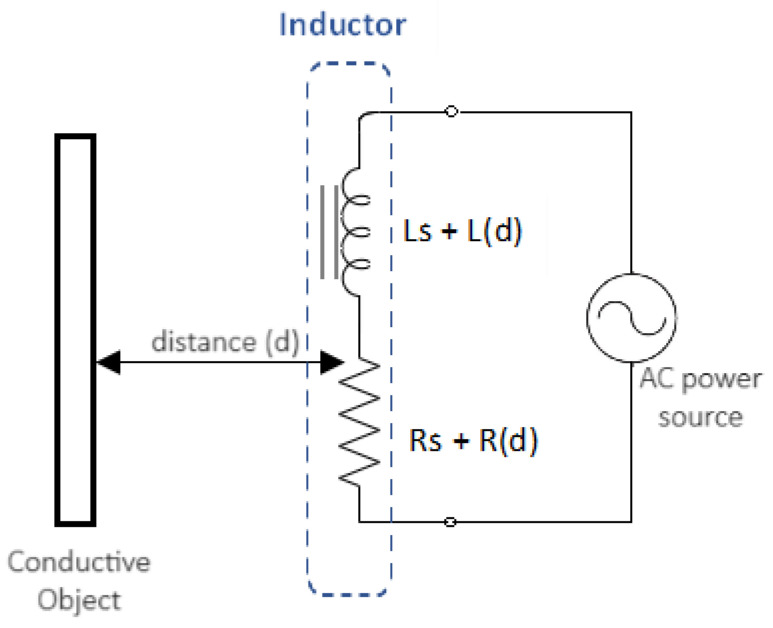
Inductor modeling with eddy current principle.

The eddy current coil can be modeled as an inductor *L* in series with a resistor $R_{s}$ (see Figure 1), where the resistor $R_{s}$ signifies the parasitic resistance of the coil. When an alternating current (AC) passes through the inductor, it generates an alternating magnetic field. In the presence of a conductive object near this magnetic field, the eddy current effect induces a change in $L_{s}$ and $R_{s}$, which vary as a function of the distance (d) between the inductor and the object, as well as the characteristics of the conductor.

However, relying solely on an inductor to produce an alternating magnetic field demands a significant amount of power. As a solution, the power consumption is lowered by adding a parallel capacitor, forming an LC resonator. By carefully selecting the values of the coil and capacitor, the circuit is tuned to resonate at a specific frequency.

The LC circuit resonates at a frequency ($f_{0}$) given by Equation (1):

(1) $$f_{0}=\frac{1}{2\pi \sqrt{LC}}$$

where ($f_{0}$) is the resonance frequency, (*L*) is the inductance, (*C*) is the capacitance, and (π) is the mathematical constant Pi (approximately 3.14159).

When modeling an LC resonator, one common topology is the parallel R-L-C model, in which the resistor (*R*), inductor (*L*), and capacitor (*C*) components are all connected in parallel. For the purpose of simplifying the calculations related to the coil’s amplitude, this parallel electrical model is preferred. In this model, the inductive component (*L*) represents the probe’s coil, which is part of the eddy current probe assembly. The capacitive component (*C*) is typically a discrete capacitor that is selected or tuned to set the desired resonant frequency of the circuit. The resistive element (*R*) accounts for the parasitic losses, including the inherent resistance of the coil’s windings and other inefficiencies that contribute to energy dissipation.

In an RCL parallel resonator, the detection of changes in a test object is facilitated through two primary methodologies: by tracking shifts in the resonant frequency and by examining fluctuations in parasitic resistance. The resonant frequency of the circuit is sensitive to variations in inductance and capacitance. Introducing a test object into the resonator’s influence can cause a change in the coil’s inductance, leading to a detectable alteration in the resonant frequency. This change can be quantitatively analyzed to reveal the object’s position or other related characteristics.

On the other hand, it also offers a means to monitor the parasitic resistance of the circuit, which includes all non-ideal resistive losses, including those caused by the test object through mechanisms like eddy currents. Since the parasitic resistance influences the energy dissipation in the circuit, it affects the decay rate of the oscillations after excitation. By employing a feedback loop that continuously monitors the amplitude of the oscillations in the LC circuit, changes in the decay rate can be observed. These changes are indicative of variations in the parasitic resistance. The feedback loop adjusts either the excitation signal or the measurement parameters in real time based on the measured decay rate. An increase in the decay rate suggests an increase in parasitic resistance, and, conversely, a decrease in the decay rate suggests a decrease in parasitic resistance. Therefore, by analyzing the decay rate of the oscillations, the sensor can measure the parallel resistance of the LC circuit, which also includes the contributions from the parasitic losses from eddy currents. Since the eddy current induced in a conductor is influenced by factors such as its size, composition, and distance from the coil, both the parasitic resistance and the inductance of the coil fluctuate accordingly.

## 4. Experimental Methodology: EC Sensor Design

A systematic approach was followed in designing an EC sensor to meet the specific specifications and performance criteria required for our application, which primarily focused on detecting corrosion in the reinforcement bars of an offshore breakwater structure.

Accordingly, this paper presents a resonant EC approach utilizing a single frequency. The detection probe, referred to as the LC probe in this paper, is an LC resonator consisting of an eddy current coil and a capacitor connected in parallel. The sensor’s development has been focused on two main objectives.

1. The sensor must evaluate the rebar condition from outside the concrete. Thus, the primary magnetic field generated should be strong enough to penetrate up to 5–6 cm (assuming that the concrete column thickness falls within this range).
2. The sensor should give meaningful information about the different levels of corrosion in the rebar.

Focusing on these main objectives, the approach to designing and implementing the sensing component (LC probe) is outlined through a series of pivotal steps as given below. The steps involved selecting suitable characteristics for the eddy current coil, followed by the selection of an appropriate capacitor to create the LC probe and subsequently testing the LC probe.

Step 1: Study the characteristics of the inductor/eddy current coil for the LC probe under predefined application requirements, considering the following.
  - Self-resonance frequency: designing a probe to operate near its self-resonance frequency (SRF) can enhance its responsiveness and can be advantageous for certain applications, potentially leading to improved performance. However, beyond the SRF, the probe’s inductive reactance diminishes, and it begins to exhibit capacitive characteristics, which affects its performance. Knowledge of the SRF is, therefore, important in designing LC probes that are effective and reliable across different operational scenarios.
  - Physical size of inductor: the physical dimensions of an inductor contribute to its overall inductance, which in turn determines the intensity of the magnetic field that it can generate. Additionally, it is a consideration with regard to managing the weight and physical profile of the sensor system, particularly for applications where the sensor needs to be compact and easily deployable.
  - Inductance value: the inductance (L) of an inductor is affected by its physical dimensions, including the number of turns in the coil, the cross-sectional area of the coil, and the core material. A larger inductor can have higher inductance, which can be advantageous in creating a stronger magnetic field or when working at lower frequencies, both of which can be beneficial for long-range sensing applications.
  - Inductor shape: the shape of the inductor in an LC sensor determines the geometry of the electromagnetic field that it generates. A circular spiral shape is ideal for proximity applications where the target moves orthogonally to the sensor plane because it generates a more symmetrical magnetic field compared to other shapes [17].
Step 2: Choose the most suitable parameters for the interpretation of corrosion conditions. This involves testing inductors to study the variations in parameters such as the parallel resistance ($R_{p}$), series resistance ($R_{s}$), inductance ($L_{s}$), and impedance (*Z*) during a frequency sweep. The selection is based on each parameter’s sensitivity to detect a conductive object (rebar) from a distance of 5 cm.
Step 3: Determine the operating frequency for the LC probe oscillation. This was determined by analyzing the frequency range that maximized the variation in the selected inductor’s parameters (from Step 1) to optimally reflect the corrosion condition under predefined test conditions.
Step 4: Once the frequency of operation and inductance are chosen, select a suitable capacitor value for the LC probe. This is done by substituting the inductor’s inductance value at the selected operating frequency range from Step 3 and calculating (using Equation (1)) the capacitor value so that the LC resonator’s resonance falls within the same frequency range.
Step 5: Integrate the LC probe with an inductance-to-digital converter to test the digital output of the final sensor design.

Theoretically, for an EC sensor, a lower resonance frequency in the LC resonator circuit is preferable when deeper inspection depths are required. To facilitate this, a higher inductance value for the inductor is desirable, as per the fundamental resonance equation of an LC circuit (Equation (1)).

To assess various possibilities, we tested a selection of inductors that can be categorized into two types based on their physical properties and inductance: a circular inductor with inductance in the mH range and a few planar inductors that were smaller in size and with inductance values in the μH range. We will summarize the overall results obtained for each category using the results from two inductors as follows.

**Figure 2:**
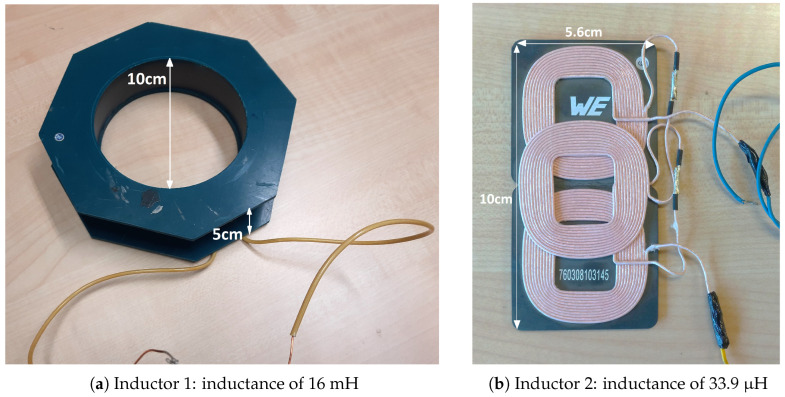
Photographs of Inductor 1 (**a**) and Inductor 2 (**b**).

Inductor 1: an inductor with a 10 cm internal diameter and inductance of approximately 16 mH (see Figure 2a).

Inductor 2: an inductor developed for wireless power applications (Part No: 760308103145) by Würth Electronics with inductance of 33.9 μH, with a series configuration of three coils (see Figure 2b) [18].

A test setup was designed to allow adjustments to the height between the inductor and the non-corroded rebar for testing at various elevations, while simultaneously maintaining a constant distance between the sensor and the non-corroded rebar during measurements. This test setup was employed to evaluate the performance of the inductors under two conditions.

**Figure 3:**
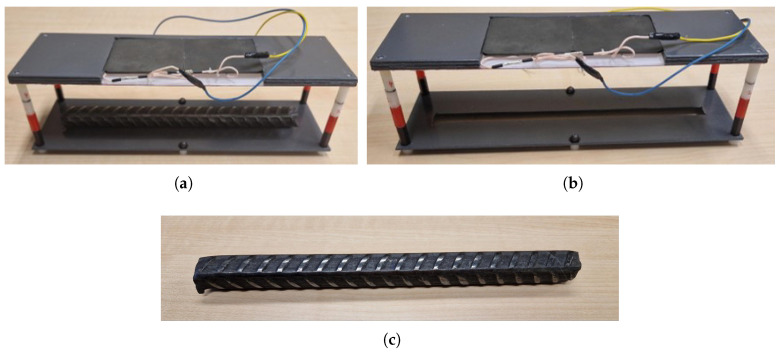
Testing condition demonstration (WO and NO) using Inductor 2 and the rebar sample S000 related to the LC probe design experiment. (**a**) Inductor 2: WO condition testing; (**b**) Inductor 2: NO condition testing; (**c**) Non-corroded rebar (S000) with 13 mm average diameter.

Condition 1:  WO condition—with object condition, where a non-corroded rebar was positioned at a 5 cm distance within the vicinity of the inductor, as shown in Figure 3a;
Condition 2:  NO condition—no object condition, where the rebar was removed from the inductor’s vicinity, as shown in Figure 3b.

Based on our application, we assume that the WO condition with a non-corroded rebar represents 0% corrosion, and the NO condition in the absence of a rebar represents 100% corrosion.

**Figure 4:**
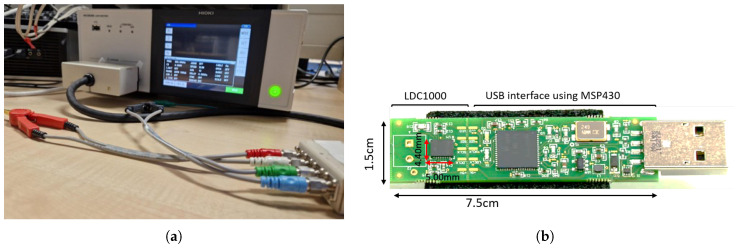
Testing equipment used during the experiment. (**a**) HIOKI IM3536 (laboratory-level testing); (**b**) LDC1000EVM device.

Following the design and implementation steps outlined in the beginning of this section, an experimental framework was established featuring a series of testing phases using two key pieces of test equipment: an LCR meter (HIOKI IM3536—Figure 4a) and an LDC1000EVM evaluation module from Texas Instruments (Dallas, TX, USA) (Figure 4b). The LCR meter was used to evaluate the inductor properties aligned with our application requirements. The main objectives were to determine the most appropriate parameters for the interpretation corrosion conditions, study the characteristics of the inductor design, and select a suitable operating frequency range for the LC probe oscillation.

Subsequently, the digital output produced by the LC probe was assessed by integrating the LC probe with the LDC1000EVM inductance-to-digital converter [19]. This test aimed to evaluate the digital output produced by the LC probe when interfaced with an LDC100x series IC, which will be incorporated into our final sensor node design.

In the following Section 4.1 and Section 4.2, the methods, results, and key conclusions from the measurements conducted with the LCR meter and the LDC1000EVM are discussed, respectively.

### 4.1. Testing Inductors’ Properties: Using LCR Meter

The inductors were positioned on the test setup with their terminals connected to the measurement probes of the LCR meter for a frequency sweep analysis under both WO and NO conditions. During the WO condition testing, the non-corroded rebar S000 was placed in the setup at a fixed distance of 5 cm from the inductor, as illustrated in Figure 3a.

In the data analysis, the percentage of variation observed at a 5 cm distance between the WO and NO conditions was evaluated across a selected set of parameters. These parameters included the series inductance ($L_{s}$), impedance (*Z*), parallel resistance ($R_{p}$), and series resistance ($R_{s}$) of the inductor. As an example, the percentage of variation for $R_{p}$ is given in Equation (2). This assessment provides a clearer insight into the frequencies that effectively represent different levels of corrosion between 0% and 100% corrosion with a better resolution.

(2) $$\Delta R_{p}\left(\%\right)=\frac{R_{p}^{\mathrm{NO}}-R_{p}^{\mathrm{WO}}}{R_{p}^{\mathrm{NO}}}\times 100$$

Here, $R_{p}^{\mathrm{NO}}$ is the no object (NO) condition and $R_{p}^{\mathrm{WO}}$ is the with object (WO) condition, where the non-corroded rebar is located in proximity to the inductor at a fixed distance. Taking into account the uncertainties of the steel bar’s slight orientation that could occur when placing the bar in the setup, several measurements were performed for the same steel bar at different times to assess the percentage of variation in the parameters.

**Figure 5:**
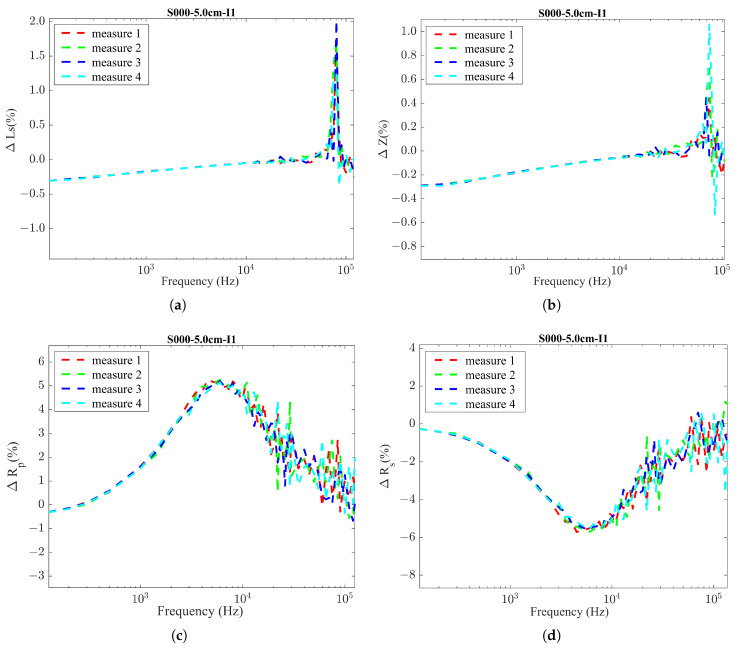
Inductor 1 testing results using LCR meter: % variations in the inductor parameters with a frequency sweep considering WO and NO conditions. (**a**) % variation in inductance (*L_s_*); (**b**) % variation in impedance (*Z*); (**c**) % variation in parallel resistance ($R_{p}$); (**d**) % variation in series resistance (*R_s_*).

Given the results in Figure 5, only the parameters $R_{p}$ and $R_{s}$ (which are closely related and provide the same information) show a significant percentage of variation.

**Figure 6:**
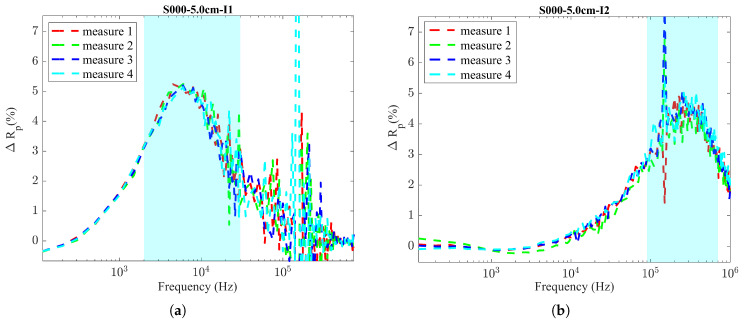
Comparison of percentage of variation in $R_{p}$ ($\Delta R_{p}$%). (**a**) Inductor 1; (**b**) Inductor 2.

On the other hand, *L_s_* and *Z* did not seem to be suitable for the analysis. Therefore, we chose the parameter $R_{p}$ as a suitable parameter to interpret the different corrosion levels. This analysis was conducted for both Inductor 1 and Inductor 2, and a comparison between the percentages of variation in $R_{p}$ between the two inductors is illustrated in Figure 6. The comparative analysis confirmed that both inductors demonstrated a similar percentage of variation in $R_{p}$ under the WO and NO conditions in a specific frequency range. These favorable frequency bands were identified for each inductor as 2–30 kHz for Inductor 1 and 90–700 kHz for Inductor 2. Even though we observed similar variations for both inductors, they are given as relative values compared to other tested frequencies. The actual quantified values provided by these two inductors when formed as LC probes for WO and NO conditions might present a different scenario. It is important to mention that we expect to have a considerably larger difference in $R_{p}$ under the WO and NO conditions, representing 0% and 100% corrosion, as there should be a sufficient resolution to represent intermediate corrosion levels.

Upon identifying the favorable frequency range, as shown in Figure 6, we proceeded to select the appropriate resonance frequency for the LC resonator. This involved matching each inductor with a compatible capacitor to construct an LC probe, ensuring that the probe’s oscillation frequency fell within the chosen frequency range for each inductor, as well as the availability of standard capacitance components. The selected capacitor value for each LC probe was 2.7 nF for Inductor 1 and 22 nF for Inductor 2. While sweeping the frequency, the resonant frequency will correspond to the point where the impedance reaches a minimum for a series LC circuit or a maximum for a parallel LC circuit. Accordingly, the oscillating frequencies of each LC probe obtained experimentally were 24 kHz and ~180 kHz, respectively. These resonance frequency values may deviate slightly from the theoretically calculated values due to the component tolerances, parasitic capacitance, and environmental conditions during testing.

The corresponding LC probes were then tested using the LDC1000EVM device to evaluate the digital output. This evaluation aimed to determine the effectiveness of the LC probes in detecting various corrosion levels, which will be discussed in the following section.

### 4.2. Inductance-to-Digital Converter: LDC1000EVM

In our final sensor solution, it was decided to integrate the LC probe with an inductance-to-digital converter to obtain its digital output value. For this purpose, we utilized the LDC100x series from Texas Instruments, which incorporates inductive sensing technology, alongside the selected LC probes.

During the sensor design phase, the LC probes were tested using the LDC1000EVM evaluation module by Texas Instruments (Dallas, TX, USA), which incorporates the LDC1000 IC [20]. The LDC1000EVM device is an inductance-to-digital converter designed for parallel resistance ($R_{p}$) and inductance (*L*) measurements. The LDC1000EVM facilitates the evaluation of the capabilities of the LDC1000 IC and the validation of sensing circuits depending on the sensing application.

**Figure 7:**
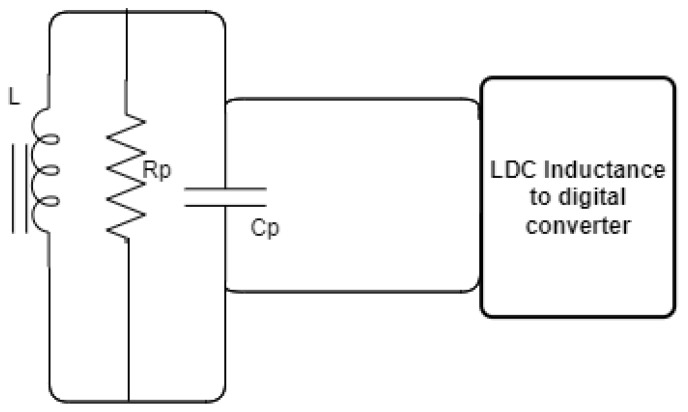
Equivalent resistance of RS in parallel with LC probe.

An inductor can be modeled as discussed in Section 3. Figure 7 illustrates how the connection between the LC probe and the LDC1000EVM has been formed.

As a conductive object moves closer to or away from the coil, it changes the characteristics of the coil, which can be observed as a variation in the inductance (*L*) and in the series resistor ($R_{s}$). Since these variations are small in Rs but significant when converted into the parallel resistance ($R_{p}$), changing from a few kilo-ohms to hundreds of kilo-ohms, the LDC1000EVM measures the equivalent parallel resonance impedance ($R_{p}$) during the measurement.

The LDC1000 supports a wide range of LC combinations, with oscillation frequencies ranging from 5 kHz to 5 MHz and $R_{p}$ ranging from 798 Ω to 3.93 MΩ. The range of $R_{p}$ is typically much smaller than the maximum input range supported by the LDC1000. To enhance the resolution within the target sensing range, the LDC1000 features programmable input range capabilities, adjustable through the $Rp_{min}$ and $Rp_{max}$ registers. Consequently, it is very important to configure the $R_{p}$ range appropriately by selecting suitable $Rp_{min}$ and $Rp_{max}$ limits before initiating the measurements, to yield meaningful results. The LDC1000EVM facilitates this process by allowing the user to set these parameters for the $R_{p}$ limits through a graphical user interface that accompanies the device.

In the LDC1000, the resonance impedance is calculated from the digital output code (*Y*), as shown in Equation (3).

(3) $$R_{p}=\frac{\left(Rp_{max}\times Rp_{min}\right)}{\left(Rp_{min}\times \left(1-Y\right)+Rp_{max}\times Y\right)}$$

There are 16 bits allocated for the $R_{p}$ parameter. Considering the change in $R_{p}$ with respect to *Y* (proximity), we obtain

(4) $$\frac{dRp}{dY}=-\frac{\left(Rp_{max}-Rp_{min}\right)\times Rp^{2}}{2^{15}\left(Rp_{max}\times Rp_{min}\right)}$$

If ΔRp is the change in $R_{p}$ for 1 bit,

$$\Delta Rp=\frac{dRp}{dY}\times \Delta Y$$

where ΔY is the change in *Y* for 1 bit.

Thus, to obtain the number of bits to represent $R_{p}$, we denote this value as *nb*:

(5) $$\frac{\left(Rp_{max}-Rp_{min}\right)}{\Delta Rp}=\frac{-2^{15}\times Rp_{max}\times Rp_{min}}{Rp^{2}}$$

(6) $$nb=log_{2}\left(\frac{2^{15}\times Rp_{max}\times Rp_{min}}{Rp^{2}}\right)=15+log_{2}\left(\frac{Rp_{max}\times Rp_{min}}{Rp^{2}}\right)$$

Reducing the error of *R_p_* due to the noise of the measurement,

(7) $$log_{2}\left(\frac{2^{15}\times Rp_{max}\times Rp_{min}}{Rp^{2}}\right)=15+log_{2}\left(\frac{Rp_{max}\times Rp_{min}}{Rp^{2}}\right)-log_{2}\left(\frac{Rp_{max}-Rp_{min}}{\Delta Rp_{corrosion}}\right)$$

Here, $\Delta R_{p_{\mathrm{corrosion}}}$ is the value obtained from $\left(Rp_{\mathrm{NO}}-Rp_{\mathrm{WO}}\right)$.

However, if $\Delta Rp_{corrosion}$/$2^{nb}$ < $Rp_{\mathrm{standard}\;\mathrm{deviation}\;\left(\mathrm{WO}\right)}$, the measurement noise dominates the resolution.

In this case, the number of bits to represent the $R_{p}$ is given by

(8) $$\mathrm{Number}\;\mathrm{of}\;\mathrm{bits}\;\mathrm{to}\;\mathrm{represent}\;R_{p}\;\left(nb\right)={log}_{2}(\frac{\Delta Rp_{corrosion}}{2}\times \frac{1}{Rp_{\mathrm{standard}\;\mathrm{deviation}\;(\mathrm{WO})}})$$

#### LC Probe Testing with LDC1000EVM

The LC probes were tested using the LDC1000EVM under the same two conditions, WO and NO, using the same experimental setup (see Figure 3).

**Figure 8:**
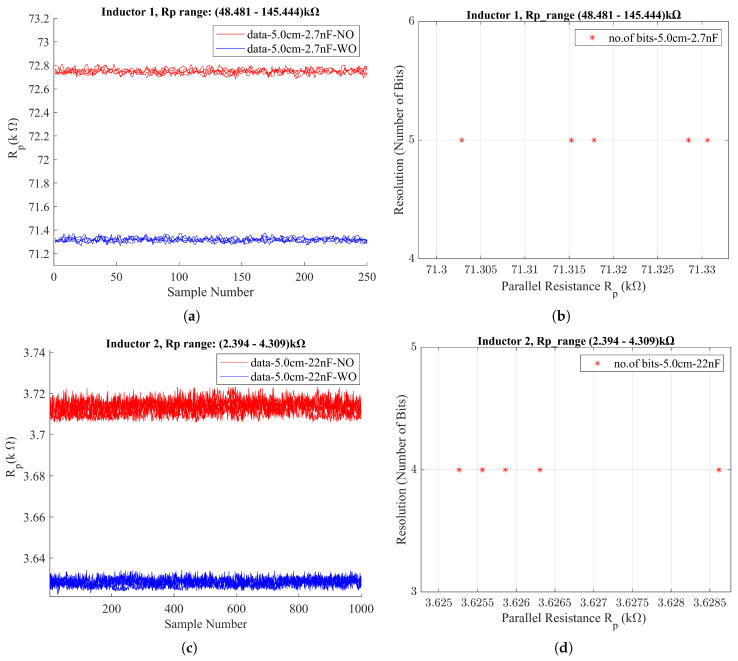
Results from LDC1000EVM for LC probes under WO (S000) and NO conditions. (**a**) $R_{p}$ value for LC probe with Inductor 1 with 2.7 nF; (**b**) No. of bits to represent corrosion for LC probe with Inductor 1 with 2.7 nF (**c**) $R_{p}$ value for LC probe with Inductor 2 with 22 nF; (**d**) No. of bits to represent corrosion for LC probe with Inductor 2 with 22 nF.

During a measurement event, a set of discrete data points is obtained, which may show small variations and sometimes include outliers, as can be seen in Figure 8a,c. To address these irregularities, several filtering steps are implemented before determining the final $R_{p}$ value. Outliers are managed using median filtering. Outliers in a dataset of 200 points per measurement were addressed using a moving median filter with a window size of 51. This method involves sliding a window across the dataset and calculating the median value for the data within each window. Each data point is then compared to its corresponding moving median. Those deviating significantly from the moving median (calculating the mean absolute deviation (MAD) within the window), beyond a certain threshold (3 × MAD), are identified as outliers. The detected outliers are then replaced using linear interpolation to ensure a more consistent and accurate representation. Subsequently, the median of this filtered dataset is selected as the final $R_{p}$ value for this particular measurement event.

The results obtained from the LDC1000EVM reveal a greater difference between the WO and NO conditions for Inductor 1, approximately 1.5 kΩ, compared to Inductor 2, which exhibits a smaller difference of around 100 Ω. These results are illustrated in Figure 8 and were achieved by selecting the best suitable $Rp_{min}$ and $Rp_{max}$ limits chosen within the LDC1000EVM for each inductor. The selection of the minimum and maximum limits for the LC probe was performed by systematically testing a series of narrow limit bands close to each other. This was performed to ensure that the measured $R_{p}$ value would be adequately bound by these limits, yet not excessively distant from either threshold, thereby optimizing the scaling resolution. The optimal band was identified through comparative evaluations, with a focus on achieving the best performance outcomes from the LDC1000EVM.

The $R_{p}$ value is affected by the distance between the sensing coil and the conductive target. As the distance increases between the coil and the conductive object, *R_p_* typically increases due to the decrease in eddy current losses [21]. Therefore, we can observe that $R_{p}$ increases in the NO condition compared to the WO condition in the results obtained for the resonators. Furthermore, the resonance frequency of the LC resonators has also affected the $R_{p}$ value in our results. A higher resonance frequency typically results in weaker eddy currents as the energy transfer at these frequencies is less efficient, particularly when there is an increased distance between the inductor and the conductive object. Therefore, considering the resonance frequency of each LC resonator, we observe differences in the *R_p_* value range: the LC resonator with Inductor 1 at 2.7 nF exhibits an *R_p_* value of around 72 kΩ, while the LC resonator with Inductor 2 at 22 nF has an *R_p_* value of 3.6 kΩ.

From the test results obtained for the inductors, it can be observed that better results are achieved (a larger difference in the $R_{p}$ value between the NO and WO conditions) with Inductor 1, in which the inductance is in the mH range. In theory, as the inductance L increases, the resonance frequency of the inductor decreases, as in the relation shown in Equation (1); this is consistent with our experimental observations.

Moreover, it has been observed that, for both inductors, the following condition holds true:

(9) $$\frac{\Delta R_{p_{\mathrm{corrosion}}}}{2^{n_{b}}}<R_{p_{\mathrm{standard}\;\mathrm{deviation}\;\left(\mathrm{WO}\right)}}$$

This implies that we will lose some bits from the allocated total number of bits for $R_{p}$ due to measurement noise (standard deviation), as represented in Equation (8).

Considering the testing results from both the LCR meter and LDC1000EVM, the following conclusions have been drawn.

1. The parameters that effectively differentiate the corrosion levels in the inductors are only $R_{p}$, $R_{s}$.
2. Inductors with lower inductance (in the µH range) tend to exhibit smaller differences in $R_{p}$ between the WO and NO conditions, indicating potentially limited sensitivity to corrosion levels.
3. Conversely, inductors with higher inductance (in the mH range) demonstrate comparatively larger differences in $R_{p}$ between the WO and NO conditions, suggesting enhanced sensitivity to corrosion levels and thus being more suitable for corrosion detection applications.
4. Selecting higher inductance for the LC probe results in a lower oscillation frequency, as evidenced by the performance of Inductor 1, which oscillated at around 24 kHz; this indicates the potential for enhanced inspection depths.

Following the above conclusions, an ad hoc inductor has been designed. This new inductor maintains similar characteristics to Inductor 1 but specifically features a reduced weight and size.

### 4.3. Ad Hoc Inductor Design Based on Experimental Results

The characteristics of the ad hoc designed inductor are tabulated in Table 1. The eddy current coil/inductor chosen for this purpose is an air-cored circular coil that is commonly used in eddy current testing applications.

**Figure 9:**
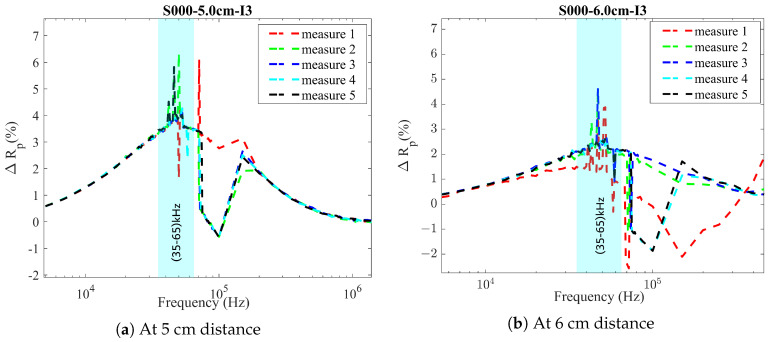
$\Delta R_{p}\left(\%\right)$ study using Inductor 3 with a frequency sweep at a distance of (**a**) 5 cm and (**b**) 6 cm (measurements from Hioki IM3536 LCR meter).

The ad hoc designed inductor is named Inductor 3 hereafter. Inductor 3 was also tested under the same procedure: the initial phase involved characterizing Inductor 3 using an LCR meter, followed by assessments using the LDC1000EVM. Additionally, the frequency range of Inductor 3 was tested at a distance of 6 cm, considering that the concrete thickness can vary between 5 and 6 cm. For both scenarios, 35–65 kHz was selected as the suitable operational frequency range of the LC probe (see Figure 9).

**Figure 10:**
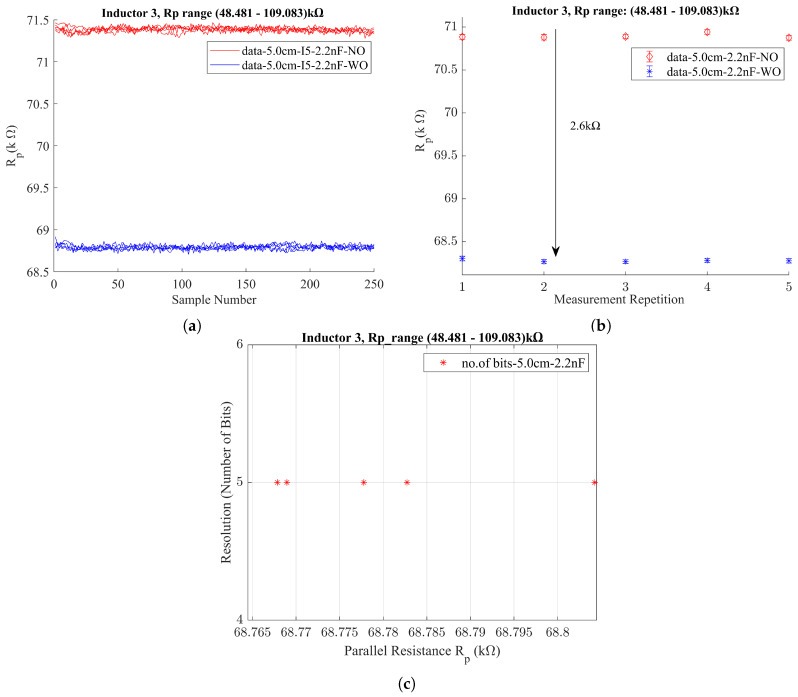
LC probe (with Inductor 3 and 2.2 nF capacitor) testing with sample S000 using LDC1000EVM. (**a**) $R_{p}$ variation. (**b**) Final $R_{p}$ value estimated for 5 measurements. (**c**) Number of bits to represent corrosion condition.

As the resonance frequency falls within this range, three capacitor values of 6.2 nF, 3.6 nF, and 2.2 nF were tested for the LC probe, each paired with Inductor 3. The experimentally obtained resonance frequencies for each pairing were 38 kHz, 48.5 kHz, and 62 kHz, respectively. Then, each LC probe configuration was assessed using the LDC1000EVM by Texas Instruments (Dallas, TX, USA). After thorough testing, the 2.2 nF capacitor was identified as the optimal selection for the LC probe. The performance of the LC probe equipped with the 2.2 nF capacitor and Inductor 3 is illustrated in Figure 10.

According to the results obtained from the LDC1000EVM, the LC probe with Inductor 3 and 2.2 nF capacitor demonstrates improved performance compared to the LC probe we tested with Inductor 1 and 2.7 nF capacitor. The difference of $R_{p}$ value could obtain around 2.6 kΩ and 5 bits to represent the corrosion condition.

**Figure 11:**
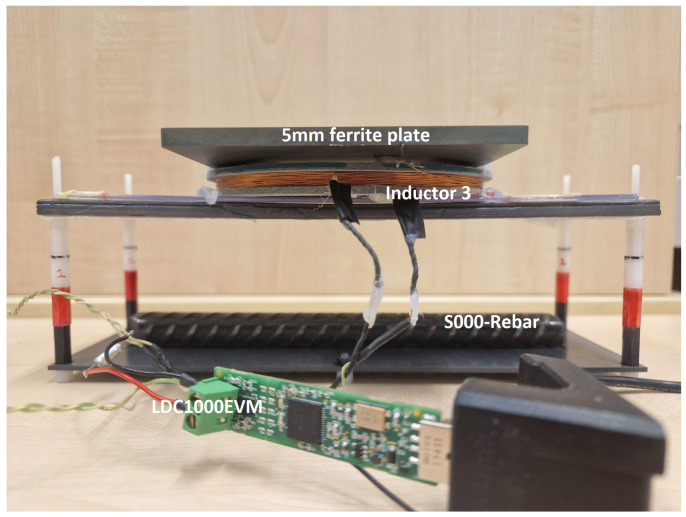
EC sensor with final LC probe and LDC1000EVM placed on the test setup.

In an effort to further refine the LC probe’s performance, a ferrite plate with a thickness of 5 mm was positioned on top of Inductor 3. This adjustment aimed to direct the magnetic field lines downward, thereby improving the focus and sensitivity of the probe. With the new modification, we achieved an $R_{p}$ difference of approximately 7 kΩ between the WO and NO conditions under the same distance conditions as before (with the sensor-to-rebar distance of 5 cm) and with an average of 6 bits to represent the corrosion condition. Based on these promising results, we finalized our LC probe design by incorporating a 5 mm ferrite plate in addition to the LC probe—Inductor 3—and the 2.2 nF capacitor, as shown in Figure 11.

Subsequently, the EC sensor, which combines the LC probe with an LDC1000 inductance-to-digital converter, will be integrated with the necessary front-end electronics, including wireless communication capabilities, to develop a complete EC sensor node. This sensor node will then be ready for field testing integration.

The final version of the LC probe was subjected to an experiment to measure the variation in the parallel resistance, $R_{p}$, with temperature fluctuations. This investigation aimed to quantify the necessary adjustments to $R_{p}$ that account for temperature variations, ensuring that the probe provides consistent and reliable readings that can be attributed solely to the corrosion levels, rather than being affected by temperature-induced changes. More details of the experiment are discussed in the following section.

### 4.4. Temperature Experiment

**Figure 12:**
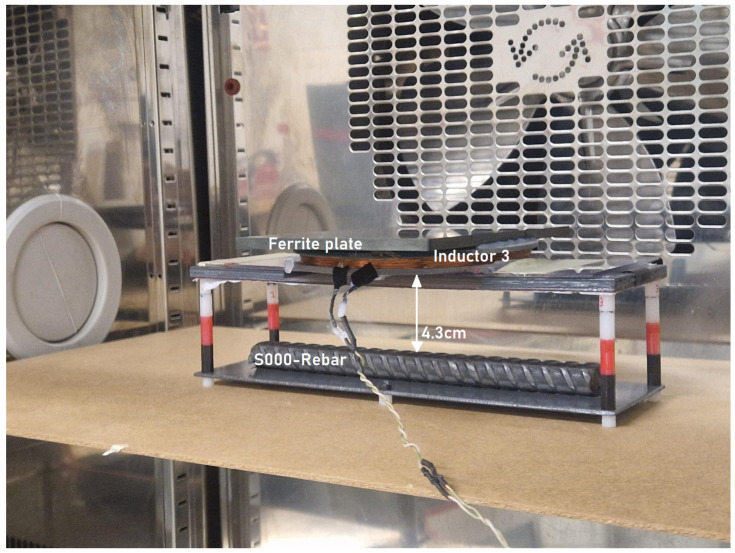
Experimental setup inside the thermal chamber; study of $R_{p}$ value under WO (S000) condition.

The test setup with the inductor and the 5 mm ferrite plate was placed inside a thermal chamber (VT 4011 from Vötsch Industrietechniks) (refer to Figure 12).

During the temperature experiment, the capacitor in the LC probe and other electronics were placed outside the chamber. The variation in the parallel resistance ($R_{p}$), as a function of the temperature, was observed across a range extending from 0 °C to 45 °C. The measurements were carried out at every 5 °C interval within this range, allowing the temperature to stabilize before the measurements. The experiment was conducted under the with object (WO) condition, which we assumed to be closer to the real scenario, with the hypothesis being that the temperature variations affecting both the rebar and the sensor would be similar. However, this assumption has its limitations, as it may not fully reflect the real temperature conditions. Given that, in practical applications, the rebars are embedded within a concrete structure, this can lead to temperature gradients or delays in temperature equilibration between the rebar and the sensor placed externally.

The first part of the experiment was conducted with the sensor placed 5 cm away from the rebar. This experiment was conducted bidirectionally, with temperature variations being tested both from 0 °C to 45 °C and in reverse, from 45 °C to 0 °C. This approach helped to account for any hysteresis effects in the $R_{p}$ response due to temperature changes, ensuring that the $R_{p}$ value’s dependence on the temperature was consistent and reproducible regardless of the direction of the temperature change. The relationship between *R_p_* and the temperature was nonlinear, best described by a quadratic model. The results indicated similar fitting in both directions, with *R_p_* decreasing as the temperature increased. Based on the temperature fitting data obtained for temperature compensation in the temperature range of 20–25 °C (considered the normal laboratory ambient temperature range), it is estimated that an approximately 155 Ω variation in parallel resistance ($R_{p}$) can be expected for every 1 °C change in temperature.

**Figure 13:**
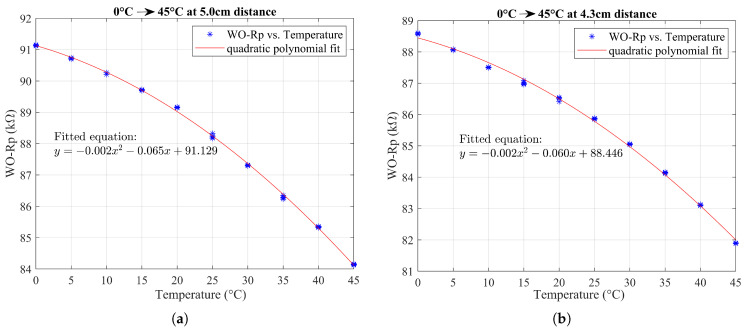
Comparison of $R_{p}$ response to temperature variations (**a**) at 5 cm distance; (**b**) at 4.3 cm distance.

Following this, the study was extended to a reduced distance of 4.3 cm between the sensor and the rebar. Comparing both distances, the results showed that the coefficients for the temperature fitting were very similar (see Figure 13), demonstrating a consistent thermal response regardless of the distance.

Considering the quadratic fitting equation $y=ax^{2}+bx+c$, where *a*, *b*, and *c* are the coefficients of fitting, Table 2 presents the fitting coefficients obtained from each group of temperature experiments.

The experimental results demonstrate that the sensor–rebar distance is not a significant factor affecting the thermal relationship with $R_{p}$, which simplifies the temperature compensation since similar behavior is observed even when variations in sensor placement occur.

## 5. Concrete Experiment with EC Sensor

Our EC sensor’s performance was tested under conditions closely simulating reinforced corrosion detection scenarios. An experiment was designed to assess the rebar conditions within a high-performance concrete (HPC) block measuring 15 × 15 × 15 cm. A hole was created inside the block to accommodate the rebar. The experiment aimed to test the sensor’s performance across different corrosion levels by mechanically degrading the rebar to simulate corrosion. The rebar sample (S001) used in the experiment was 50 cm long. The core, representing the cylindrical section of the bar, had a diameter of approximately 11.5 mm. Additionally, the corrugations on the surface of the rebar, which varied in height, measured between 0.5 mm and 1 mm.

At first, measurements were taken with the rebar at its initial, non-degraded state. Subsequently, uniform material degradation was applied along the rebar to simulate various corrosion levels. During the first degradation step, the material was selectively removed to achieve an approximately 3% reduction in the diameter of the cylindrical section of the rebar, resulting in an additional amount of material being removed due to the presence of surface corrugation.

**Figure 14:**
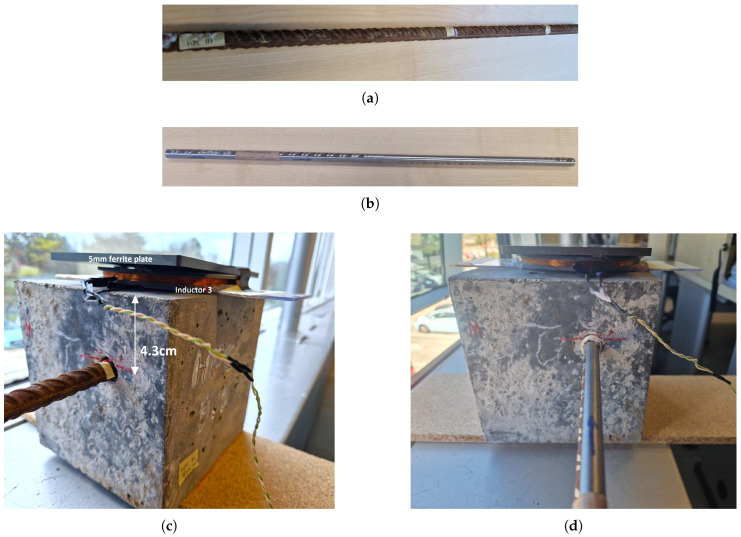
The rebar (S001) measurements with the mechanical degradation process. (**a**) Sample: S001. No degradation; (**b**) Sample: S001 After first degradation; (**c**) Concrete test before any degradation; (**d**) Concrete test after first degradation.

The testing setup, shown in Figure 14c,d, involved placing the rebar inside the concrete block and positioning the sensor on the outer surface during the measurements.

The measured distance between the sensor and the top of the rebar was 4.3 cm. Throughout the experiment, efforts were made to keep the bar orientation unchanged. In real conditions, the rebar remains fixed. To closely replicate real-world conditions and ensure the consistency and relevance of our methodology, we maintained the same orientation of the rebar.

The main objective of this experiment was to establish a definitive correlation between the fluctuations in parallel resistance ($R_{p}$), as detected by our EC sensor, and the weight loss of the rebar, which is indicative of varying levels of corrosion. To ensure that the variations in $R_{p}$ were attributed solely to material loss, the rebar was kept at a consistent elevation relative to the sensor. As we induced uniform degradation along the length of the rebar, a relevant shift in the mid-axis level was expected due to the material loss occurring uniformly around the rebar once it was placed within the concrete. However, we controlled this by keeping the central axis at the same height throughout the measurement process after each degradation step. In actuality, in the case of uniform corrosion, the central axis of the rebar generally remains in the same position during the corrosion process, because the corrosion products, which can include rust and other compounds, often fill the spaces left by the corroded metal. This means that although the rebar is losing its original material to corrosion, the volume may appear to be conserved due to the accumulation of these products. By simulating this aspect in our experimental setup, we aimed to measure the changes in $R_{p}$ that would directly correspond to the actual loss of material, as would be the case in a natural corrosion process.

### Results and Discussion

The mechanical degradation simulation, aimed at replicating reinforcing bar corrosion, yielded results across four distinct stages of degradation. The first was 0% corrosion, where we measured the non-corroded S001 bar without any degradation. This was followed by the first degradation stage, where the S001 bar was degraded with 10% weight loss from its initial weight. Afterwards, the second degradation stage was assessed, with the 3% weight loss of the bar; finally, 100% corrosion was considered, where we measured it without the presence of the rebar to simulate complete corrosion to represent 100% corrosion.

After the first degradation, the results showed a difference of approximately 1.2 kΩ in the $R_{p}$ value compared to the non-degraded state. This corresponds to an average of 11% weight loss and a 14% change in the $R_{p}$ value. Since the difference in $R_{p}$ between the two states is quite high, there is potential to detect lower percentages of degradation than the observed 11%. Therefore, the second degradation was performed as we lost approximately 3% of the material, with a reduction in diameter of around 0.17 mm.

**Figure 15:**
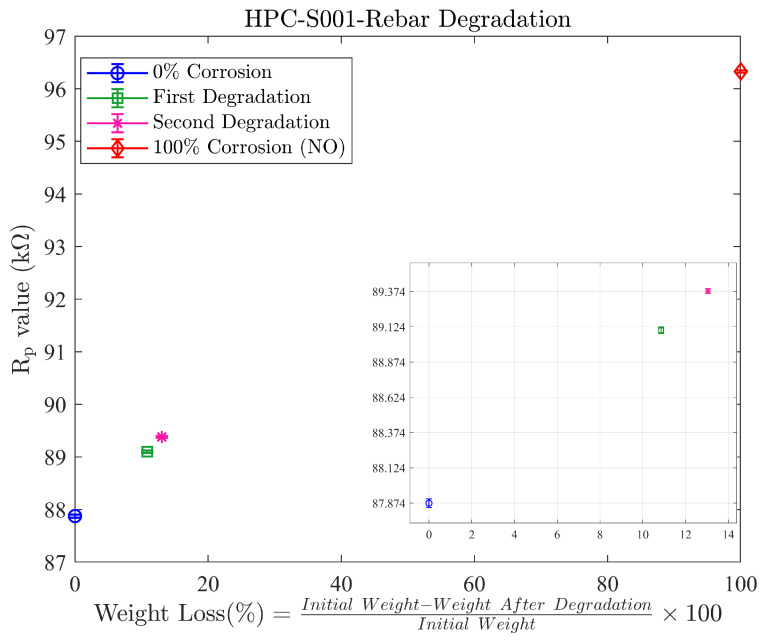
$R_{p}$ value vs. corresponding weight during the corrosion experiment with rebar S001.

As the results shown in Figure 15, we obtained a $R_{p}$ range of 8.45 kΩ between 0% and 100% corrosion, providing a substantial range to represent various degrees of corrosion.

After the second degradation step, there was a 275 Ω increase in the *R_p_* value compared to the first degradation, corresponding to 3% weight loss in the rebar. This means that the sensor is capable of detecting 3% weight loss, accounting for material loss around the whole rebar, resulting in less than a 0.2 mm (approximately 0.17 mm) reduction in diameter.

**Figure 16:**
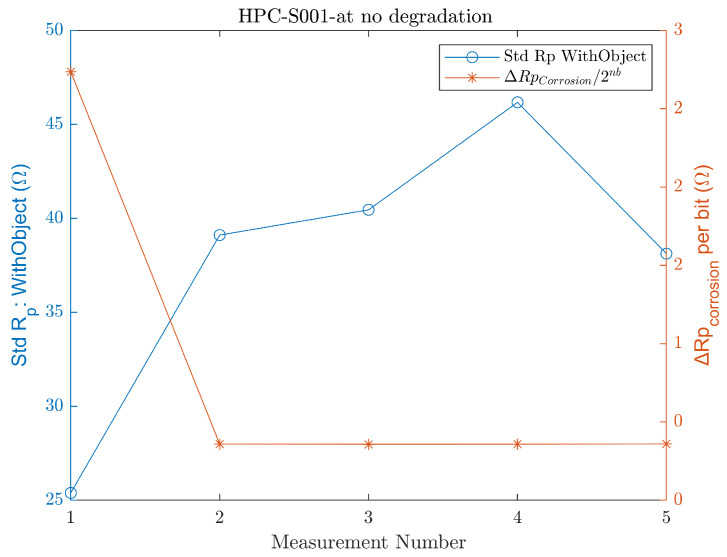
Change in $R_{p}$ for 1 bit of the register allocated for $R_{p}$ and the standard deviation of 5 consecutive measurements performed for non-corroded S001.

The amount of the *R_p_* increase observed during the second degradation test is substantial enough to distinguish another level of corrosion without being affected by the standard deviation of the measurements. Typically, after handling outliers, the standard deviation of the measurements normally ranges between 20 Ω and 60 Ω, as shown in Figure 16.

Figure 16 presents the standard deviation of the measurements conducted at 0% corrosion (no-degradation) during the concrete test, which can be generalized to the performance of the final version of the EC sensor.

Furthermore, a comparison between the standard deviation of the measurements and the Δ$R_{p}$ that is the $R_{p}$ value represented per 1 bit under the test conditions is presented, indicating that, typically, the standard deviation of the measurements influences the resolution, as discussed in Section 4.2. Hence, Equation (8) shows the relationship that governs the number of bits to represent the measured $R_{p}$ values.

Considering the impact of the temperature on $R_{p}$, our experimental results showed that, for each 1 °C deviation within the 20–25 °C range, the $R_{p}$ values need to be corrected by 155 Ω. Although the laboratory conditions were relatively stable, with minor temperature variations, in real-world scenarios, where the environmental conditions are far more variable, temperature-compensated data are essential to account for the influence of temperature fluctuations on the $R_{p}$ measurements for the relative assessment of corrosion conditions.

The uniform mechanical degradation cycles employed in our experiments are indeed a simplification of the natural corrosion process, primarily aimed at testing our sensor’s performance under the accelerated wear that materials might experience in certain offshore conditions. This approach, while simulating uniform corrosion along the rebar (S001) at different stages, does not fully capture the complex chemical interactions and environmental factors inherent in natural corrosion. Hence, any effect on $R_{p}$ influenced by a variety of environmental conditions, such as humidity and the presence of corrosive agents, has not been accounted for. The synergistic effects of these parameters and their fluctuations over long periods, as they occur in nature, present a challenge for the laboratory simulation. Additionally, the rate of degradation in our simulations may not reflect the typically slower pace of natural corrosion, as the material degradation limitations and time are often condensed in a laboratory setting for feasibility.

## 6. Conclusions and Future Works

This paper details the development and application of a single-frequency eddy current (EC) sensor employing an LC resonator for the non-destructive evaluation of corrosion levels within concrete-embedded reinforcing bars. The sensor’s design and experimental validation process utilized a comprehensive methodology to study the optimal LC resonator characteristics, aiming to develop a system capable of detecting changes in the rebar condition from a distance of 5–6 cm outside the concrete structure, as per the application requirements. Following the experimental methodology, the EC sensing design integrates an LC probe with a circular air-cored inductor of approximately 3 mH inductance and a 2.2 nF capacitor connected in parallel, along with a 5 mm ferrite plate placed on top of the inductor.

The temperature effect on the sensor’s output (*R_p_*) was evaluated using a temperature experiment under conditions in which the eddy current coil was positioned facing the S000 rebar at a 5 cm distance. The change in *R_p_* per 1 °C in the temperature range of 20–25 °C was recorded as 155 Ω. The temperature study results showed that the sensor–rebar distance was not a significant factor affecting the thermal relationship with $R_{p}$.

Through the systematic simulation of uniform corrosion via mechanical degradation cycles, we have demonstrated the sensor’s capability to accurately quantify material loss with a resolution of approximately 0.17 mm.

While the results are promising under laboratory conditions, it is acknowledged that the mechanical degradation approach employed does not fully replicate the complex nature of real-world corrosion. Therefore, further research is required to incorporate environmental factors to enhance the sensor’s practicality under real offshore operating conditions. In future work, we aim to enhance the validity of our findings by testing the sensor’s performance under conditions that more accurately reflect real-world applications. To achieve this, the sensor will be integrated into a concrete block with real rebar conditioning (a real reinforced structure block) and tested under offshore environmental conditions. The next step will help us to identify any potential performance differences between laboratory and field conditions and refine the sensor design accordingly.

## Figures and Tables

**Table 1:** Ad hoc inductor characteristics.

Characteristic	Value
Inductor inner diameter	10 cm
Wire diameter	0.3 mm
Number of turns	110
Self-resonance frequency (tested using LCR meter HIOKI IM3536)	350 kHz
Inductance in the 35–65 kHz frequency range (tested using LCR meter HIOKI IM3536)	3 mH

**Table 2:** Quadratic fitting coefficients obtained in temperature experiment.

Control Experiment	Distance (cm)	Temp. Cycle (°C)	Coeff. *a*	Coeff. *b*
1	5	0–45	−0.002	−0.065
2	5	45–0	−0.002	−0.067
3	4.3	0–45	−0.002	−0.060

## Data Availability

Data are contained within the article.

## Abbreviations

The following abbreviations are used in this manuscript:

AC	Alternating Current
ECT	Eddy Current Testing
EC	Eddy Current
EMF	Electromotive Force
HCP	Half-Cell Potential
HPC	High-Performance Concrete
LPR	Linear Polarization Resistance
MAD	Median Absolute Deviation
NDT	Non-Destructive Testing
NO	No Object
OCP	Open Circuit Potential
PV	Photovoltaic
*R_p_*	Parallel Resistance
Rebar	Reinforcing Bar
SRF	Self-Resonance Frequency
SP	Surface Potential
UT	Ultrasonic Testing
WO	With Object
